# Duration of streptozotocin-induced diabetes differentially affects p38-mitogen-activated protein kinase (MAPK) phosphorylation in renal and vascular dysfunction

**DOI:** 10.1186/1475-2840-4-3

**Published:** 2005-03-05

**Authors:** Hongmei Chen, Sachin Brahmbhatt, Akanksha Gupta, Avadhesh C Sharma

**Affiliations:** 1 Cardionome Laboratory, Department of Pharmaceutical Sciences, College of Pharmacy North Dakota State University, Fargo, ND 58105, USA

**Keywords:** NIDDM, thoracic aorta, kidney cortex, kidney medulla, blood flow, nitric oxide synthase, endothelin-1, signaling

## Abstract

**Background:**

In the present study we tested the hypothesis that progression of streptozotocin (STZ)-induced diabetes (14-days to 28-days) would produce renal and vascular dysfunction that correlate with altered p38- mitogen-activated protein kinase (p38-MAPK) phosphorylation in kidneys and thoracic aorta.

**Methods:**

Male Sprague Dawley rats (350–400 g) were randomized into three groups: sham (N = 6), 14-days diabetic (N = 6) and 28-days diabetic rats (N = 6). Diabetes was induced using a single tail vein injection of STZ (60 mg/kg, I.V.) on the first day. Rats were monitored for 28 days and food, water intake and plasma glucose levels were noted. At both 14-days and 28-days post diabetes blood samples were collected and kidney cortex, medulla and aorta were harvested from each rat.

**Results:**

The diabetic rats lost body weight at both 14-days (-10%) and 28-days (-13%) more significantly as compared to sham (+10%) group. Glucose levels were significantly elevated in the diabetic rats at both 14-days and 28-days post-STZ administration. Renal dysfunction as evidenced by renal hypertrophy, increased plasma creatinine concentration and reduced renal blood flow was observed in 14-days and 28-days diabetes. Vascular dysfunction as evidenced by decreased carotid blood flow was observed in 14-days and 28-days diabetes. We observed an up-regulation of inducible nitric oxide synthase (iNOS), prepro endothelin-1 (preproET-1) and phosphorylated p38-MAPK in thoracic aorta and kidney cortex but not in kidney medulla in 28-days diabetes group.

**Conclusion:**

The study provides evidence that diabetes produces vascular and renal dysfunction with a profound effect on signaling mechanisms at later stage of diabetes.

## Introduction

Diabetes is a complex and multifarious group of disorders characterized by hyperglycemia that has reached epidemic proportions in the present century. Infection is a leading cause of morbidity and mortality among the diabetic population [1]. Diabetes is associated with vascular and renal dysfunction characterized by hypertension, dyslipidemia, microalbuminuria, macroalbuminuria and glomerular mesangial expansion [2,3].

Mitogen-activated protein kinases (MAPKs) are implicated in the etiology of diabetes [4,5]. MAPKs are serine-threonine protein kinases involved in cell survival, proliferation and apoptosis [6]. Three MAPK subfamilies have been well characterized: extracellular signal regulated kinase 1 and 2 (ERK1/2), c-jun N-terminal kinases (JNK) and p38-MAPK [7]. ERK1/2 is involved in the growth response of cell while p38-MAPK and JNK are associated with cellular response to stress [7], inflammation [8] and vasoactive mediators such as endothelin-1 (ET-1) [9]. Also p38-MAPK activation stimulates inducible nitric oxide synthase (iNOS) expression in serum-deprived RAW 264.7 cells [10]. These observations suggest that signaling mechanisms can regulate various vasoactive molecules and vice-versa. However it is still not known if progression of diabetes produces a time dependent activation of p38-MAPK in vascular blood vessels and kidneys. We speculate a differential regulation of p38-MAPK and ERK1/2 in thoracic aorta and kidneys depending upon the duration and severity of diabetes.

We have earlier demonstrated that diabetes during coronary artery bypass grafting, and chronic peritoneal sepsis produced an imbalance in the myocardial and systemic ET-1 and nitric oxide (NO) profiles [11,12]. However, the profile of ET-1 during progression of diabetes is unclear. Diabetes, both *type 1 *and *type 2*, is associated with decreased NO bioavailability [13,14]. Conflicting reports (i.e. increased, unchanged and decreased) exist regarding the state of inducible nitric oxide synthase (iNOS) and endothelial NOS (eNOS) during diabetes [15,16]. We have earlier reported that elevation of ET-1 and NO mechanisms, either systemically or locally in the myocardium, correlated well with the development of myocardial dysfunction during sepsis [11] and make the heart susceptible to myocardial injury [17]. We anticipate that the duration of hyperglycemia would differentially modulate systemic and renal ET-1 and NO mechanisms.

We hypothesize that progression of diabetes [mild (14-days) to moderate (28-days)] would produce renal and vascular dysfunction that correlate with altered p38-MAPK phosphorylation in kidneys and thoracic aorta. Therefore, the specific aims of the study are to characterize the progression of STZ-induced renal and vascular dysfunction at 14- and 28-days; and to examine if the progression of STZ-induced diabetes would alter the biosynthesis and activation of ET-1, NO and the phosphorylation of p38-MAPK and ERK1/2 in thoracic aorta, kidney cortex and kidney medulla.

## Materials and methods

Male Sprague-Dawley rats (Harlan, Indianapolis, IN) weighing 350–400 g were used in the study. The rats were acclimatized to the laboratory conditions for at least 7 days following their arrival. All animal experiments were conducted in compliance with humane animal care standards outlined in the *NIH Guide for the Care and Use of Experimental Animals*, and were approved by the Institutional Animal Care and Use Committee of North Dakota State University.

### Experimental Protocol

All animals were age-matched at the onset of the study. The rats were randomly divided into three groups: control, 14-days and 28-days diabetic rats (n = 6 for each group).

#### Induction of diabetes

Diabetes was produced by a single tail vein injection of streptozotocin (STZ; 60 mg/kg, I.V.) [15]. Diabetes was confirmed by blood glucose estimation (Glucometer Elite^®^, Bayer Corporation, IN) 2 days after STZ-injection. Diabetes was confirmed if blood glucose > 200 mg/dL. The animals were not given insulin supplementation. Food intake and water intake were examined everyday after STZ-injection.

The non-diabetic control rats received an injection of 1 mL/kg saline. 14-days and 28-days post-STZ administration recordings of systemic hemodynamics, carotid blood flow, and renal arterial blood were done. Arterial blood was collected in plastic tubes containing EDTA (1 mg/mL) and heparin (5 units/mL) to determine the plasma concentration of ET-1, NO by-products (NOx) and creatinine. The animals were euthanitized by pentobarbital (100 mg/kg, I.P.), the thoracic aorta and kidney were harvested. The concentration of ET-1, NOx, creatinine and protein expression of preproET-1, iNOS, eNOS, phosphorylated p38-MAPK (pp38-MAPK), total p38-MAPK, phosphorylated ERK, total ERK were determined in the thoracic aorta, kidney cortex and kidney medulla obtained from each animal.

### Surgical protocol

Separate group of animals (N = 6) were used for hemodynamic study. On the day of experiment, the rats were anesthetized with an intraperitoneal injection of pentobarbital sodium (Nembutal^®^, Abbott; 50 mg/kg).

#### Common carotid arterial blood flow measurement (acute)

Under pentobarbital (50 mg/kg I.P.) anesthesia, rats were placed in dorsal recumbency and through a midline cervical incision, the left common carotid artery was identified and carefully separated from adhering connective material. The carotid artery was cleared from the vagal nerve and a Transonic^® ^flow probe (0.3 mm 1RB, Transonic Systems Inc., Ithaca, NY) was placed carefully around the artery. The probe was manually positioned so that the artery was centered within its window and then probe was held in position. Through a sterile 10 CC syringe loaded with sterile K-Y brand lubricating jelly (Johnson & Johnson, Arlington, TX), the acoustical window within the flow probe was filled while avoiding any air bubble.

#### Renal arterial blood flow measurement (acute)

Under pentobarbital anesthesia, rats were place in dorsal recumbency, and through a midline abdominal skin incision the left renal artery was carefully separated from renal vein and a 0.5 VB Transonic^® ^flow probe was placed around it. The probe was manually positioned so that the artery is centered within the window and the probe was held in position. The acoustical window within the flow probe was filled with K-Y brand lubricating jelly (Johnson & Johnson, Arlington, TX). Renal and carotid artery blood flow was measured using Transonic^® ^flow meter T206 attached to MP100 system of Biopac Systems Inc., CA via analog to digital conversion. The MP100 system was calibrated with minimum and maximum flow capacity of the individual probe connected to the flow meter. The sampling of data was carried out at 1000 Hz and recorded to a dedicated computer using *Acq*Knowledge™ software.

### Biochemical estimations

#### Determination of the concentration of plasma creatinine

The concentration of creatinine was determined in plasma using creatinine liquid reagents (end-point, colorimetric, DIAZYME). The blood samples immediately after collection were spun down at 5000 rpm for 10 minutes. The plasma was then decanted and stored at -20°C until the time of creatinine determination using manufacturer's instruction. Plasma concentration of creatinine (mg.dL^-1^.Kg^-1^) normalized to individual rat body weight was calculated.

#### Determination of the plasma concentration of ET-1

The concentration of ET-1 was determined in plasma. The blood samples were collected in plastic tubes containing EDTA (1 mg/mL) before euthanasia. The sample was centrifuged at 3,000 × g for 15 min at 4°C and plasma was separated and assayed for ET-1. Plasma was acidified adding an equal volume of 20% acetic acid. ET-1 like material was extracted from plasma [11] using C-18 SEP-Columns (Peninsula labs, CA). The recovery of ET from plasma was approximately 87%. Immunoassay (IA) was performed using EIA kit for ET-1 (R and D systems, Minneapolis, MN). The assay was performed in microtiter plates coated with a rat antibody to human ET-1. Diluted anti-ET-1 HRP conjugate (100 μL) (ET-1 conjugated to horseradish peroxidase) was added in each well. Standards (0.25 pg – 65 pg ET-1), parameter control (24.5 ET-1 pg/ml) or sample extract (100 μL, each) were added. The plates were covered with plate sealers and incubated for 1 hr at room temperature. The contents of each well were aspirated and washed using wash buffer provided with the kit. After the last wash, contents of each well were decanted and tetramethylbenzidine (100 μL) was added. After 30 min, stop solution (100 μL) containing 1 N HCl was added. Within 30 min of addition of the stop solution, the optical density of each well was measured using a micro plate reader at 450 (OD 450) and 620 (OD 620) nm separately. A standard curve was created and the concentration of ET-1 of each sample calculated and expressed as pg/ml of plasma.

#### Determination of the concentration of nitric oxide byproducts (NOx)

The concentration of endogenous NO_X _(nitrate + nitrite) was determined in plasma, thoracic aorta, kidney cortex and medulla. Blood (500 μL) was collected and 40 μL of heparin was added to each sample to prevent clotting. The blood sample was then decanted and stored at -20°C until the time of nitric oxide byproducts, (NO_2 _+ NO_3_) NOx, determination. To determine the NOx level of thoracic aorta, kidney cortex and kidney medulla, those tissue samples were harvested from each experimental rat and immediately homogenized with cold phosphate buffered saline (PBS) on ice, which inhibited the activity of NOS *ex vivo*. The homogenate was centrifuged (3000 × g, 5 min) and the supernatant was collected. The supernatant obtained from tissues and plasma was passed through a 1.2 μm multiscreen filter plate. Plasma NOx and tissue NOx concentration was determined by using Greiss reaction [11,15]. 6 μL of plasma was mixed with 44 μl distilled H_2_O, 20 μl 0.31 M phosphate buffer (pH 7.5) and 10 μL each of 0.86 mM NADPH (Sigma), 0.11 mM flavenidinine dinucleotide and 1.0 U/mL of nitrate reductase. NO_3 _was converted to NO_2 _by nitrate reductase (Boehriger Mannheim). Unknown tissue samples were run in duplicate. The samples were allowed to incubate for 1 hr at room temperature in the dark. Two hundred microliters of Greiss reagent [1:1 mixture of 1% sulfanilamide in 5% H_3_PO_3 _and 0.1% N-(1-naphthyl) ethyl-enediamine] were added to each well and the plates were incubated for an additional 10 min at room temperature. Absorbance was measured at 540 nm using a plate reader and converted to NOx concentration using a nitrate standard curve and expressed as μM in plasma and μmoles/g protein in tissue. Protein in the supernatant obtained from each sample was determined using standard Lowry method.

### Immunoblot Analysis

PreproET-1, iNOS, eNOS, total and phosphorylated p38-MAPK and ERK protein expressions of thoracic aorta, kidney cortex and kidney medulla were determined using standard SDS-PAGE and immunoblot technique. Briefly, thoracic aorta, kidney cortex and kidney medulla tissues were homogenized in lysis buffer and centrifuged as described by Pollack et al. [16]. The supernatants, at a final protein content of 25 μl, were loaded to the gels using a 2:1 laemmli sample buffer (62.5 mM Tris-HCl, pH 6.8, 25% glycerol, 2% SDS, 0.01% bromophenol blue and 710 mM β-mercaptoethanol). The prepared samples were electrophoresed on 10% denaturing sodium dodecyl sulfate (SDS) polyacrylamide gels. The proteins were transferred electrophoretically onto polyvinylidene difluoride (PVDF) membrane (Gelman Sciences, Pierce, Rockford, IL). Non-specific binding sites on the membrane were blocked overnight at 4°C with 5% nonfat dry milk in Tris-buffered saline containing Tween 20 (TBST, 20 mM Tris-HCl, 150 mM NaCl, 0.2% Tween 20, pH 7.4). The membranes were then probed with the primary antibody (Santa Cruz Biotechnology, Santa Cruz, CA) for 1 hr at room temperature. The primary antibodies are highly specific against the proteins studies and had no cross-reactivity with related members. After five washings in TBST, the membranes were incubated with the secondary antibody (Sigma, St. Louis, MO) for 1 hr at room temperature. Finally membranes were washed three times with TBST. The specific proteins were detected by enhanced chemiluminescence (ECL) reagent (Amersham Pharmacia Biotech). The blots were analyzed using Un-Scan-It™ software to estimate the density of the blots in pixels. Uniform loading was assessed by β-actin (Sigma) protein expression. While analyzing the data for immunoblot analysis, the beta-actin blots for each gel were analyzed first to confirm equal protein loading. Only after confirming that there was equal protein loading in the wells, as evidenced by no significant difference in pixel values of beta-actin blots, the bands for individual proteins were analyzed.

### Statistical Analyses

All the data were expressed as mean ± SEM. One-way ANOVA was performed to analyze the hemodynamic and biochemical data using SPSS software. Following a significant F value, a post hoc Students Newman Keuls test was performed for inter- and intra-group comparisons. Statistical significance was realized at p ≤ 0.05 to approve the null hypothesis for individual parameters.

## Results

### General characteristics of the animals

**Figure 1:**
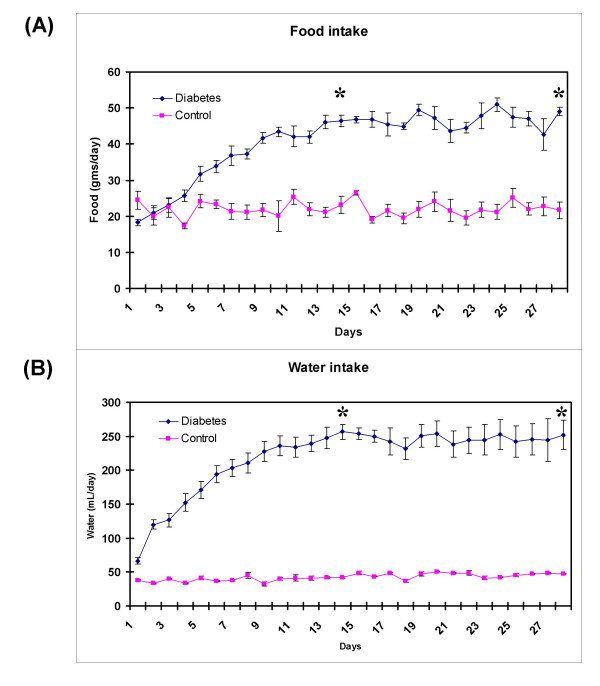
Food intake (gm/day) (A) and water intake (mg/day) (B) in control. 14-days and 28-days STZ-induced diabetes groups (N = 6 in each group); * p ≤ 0.05 Vs control group.

All control rats were freely moving in their individual cages through out the study. Although diabetic rats appeared to be lethargic and displayed restricted movements, there was no sign of infection or motor disorder in any of the rats studied. The food intake and water intake of 14-days and 28-days diabetes groups were significantly increased as compared to control group (Fig 1).

The average body weight change, blood glucose and kidney weight/ 100 g body weight in all groups are summarized in Table 1. The body weight change (%) was significantly decreased in 14-days and 28-days diabetes groups as compared to their age-matched control group. All STZ-treated rats were diabetic with mean blood glucose around 480 mg/dL. Blood glucose was significantly elevated in 14-days diabetes and 28-days diabetes groups as compared to their age-matched control group. Kidney weight normalized to body weight was significantly greater in diabetic (14-days and 28-days) rats than in control rats. Plasma creatinine concentration was increased 3-fold in 14-days diabetes group and 2-fold in 28-days diabetes group as compared to control group (Table 1). Diabetes (14- and 28-days) produced no significant change in mean arterial pressure but caused a significant decrease in heart rate (Table 1).

### Effect of STZ-induced diabetes on aortic and renal blood flow

**Figure 2:**
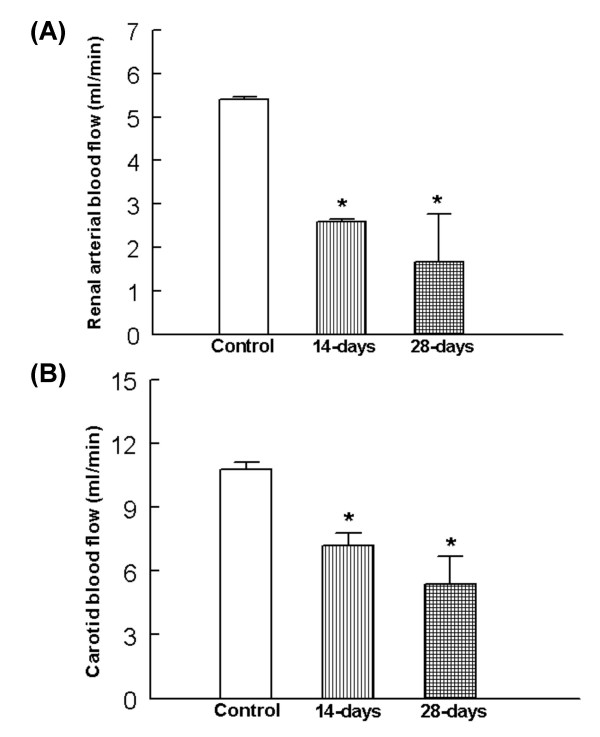
Renal blood flow (mL/min) (A) and carotid blood flow (mL/min) (B) in control, 14-days and 28-days STZ-induced diabetes groups (N = 6 in each group); * p ≤ 0.05 Vs control group.

Control groups exhibited aortic and renal blood flow 10.8 ± 0.3 mL/min and 5.4 ± 0.1 mL/min, respectively. Induction of diabetes produced a significant decrease in renal (Fig 2A) and carotid blood flow (Fig 2B) at both 14-days and 28-days as compared to control group.

### Effect of STZ-induced diabetes on the concentration of ET-1 in plasma and expression of preproET-1 in thoracic aorta, kidney cortex and medulla

**Figure 3:**
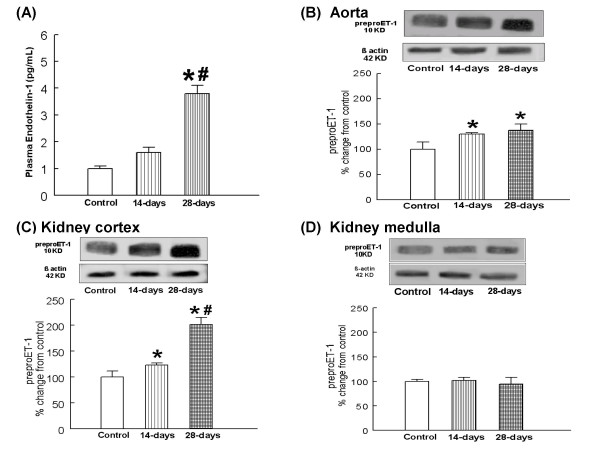
The concentration of plasma ET-1 (pg.mL^-1^) in control, 14-days and 28-days STZ-induced diabetes groups (A) (N = 6 in each group). Effect of duration (14-days and 28-days) of STZ-induced diabetes on the expression of preproET-1 protein in thoracic aorta (B), in kidney cortex (C) and in kidney medulla (D) and % change in densitometric units of preproET-1 (N = 3 in each group); * p ≤ 0.05 Vs control group, # p ≤ 0.05 Vs 14-days diab group.

To determine the effect of duration of diabetes (14-days to 28-days) on aortic and renal ET-1 biosynthesis we determined the concentration of ET-1 in plasma and expression of ET-1 precursor, preproET-1 in thoracic aorta, kidney cortex and kidney medulla. A significant elevation in plasma ET-1 concentration was observed in 28-days diabetes as compared to 14-days diabetes and control groups (Fig 3A). We did not find any significant change in the plasma concentration of ET-1 in 14-days diabetes group as compared to control group. The expression of preproET-1 was significantly increased in thoracic aorta in 14-days and 28-days diabetes groups as compared to control group (Fig 3B). We also found a significant increase in the expression of preproET-1 in kidney cortex in 14-days diabetes groups as compared to control group. A significant increase in the protein expression of preproET-1 of kidney cortex was observed in 28-days diabetes group as compared to 14-days diabetes and control groups (Fig 3C). Induction of diabetes did not produce any change in the expression of preproET-1 in kidney medulla at 14-days and 28-days diabetes groups as compared to control group (Fig 3D).

### Effect of STZ-induced diabetes on the concentration of NOx and expression of eNOS and iNOS

**Figure 4:**
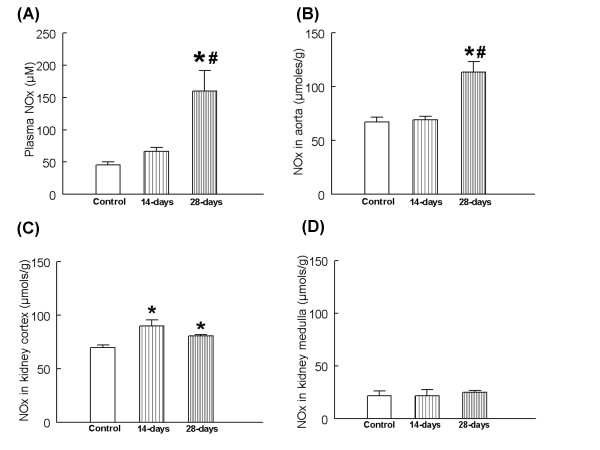
Effect of STZ-induced diabetes on the plasma (A), thoracic aorta (B), kidney cortex (C) and kidney medulla (D) concentration of nitric oxide byproducts (NOx) in control, 14-days and 28-days diabetic rats. (N = 6 in each group); * p ≤ 0.05 Vs control group, # p ≤ 0.05 Vs 14-days diab group.

A significant increase in the concentration of NOx in plasma of 28-days diabetes group as compared to the control and 14-days diabetes groups was observed (Fig 4A). The NOx level was also elevated in thoracic aorta of 28-days diabetes group as compared to the control and 14-days diabetes groups (Fig 4B). In kidney cortex, we also found that NOx level was significantly increased in 14-days and 28-days diabetes groups as compared to control group (Fig 4C). But we did not find any significant change in kidney medulla of 14-days and 28-days diabetes groups (Fig 4D).

**Figure 5:**
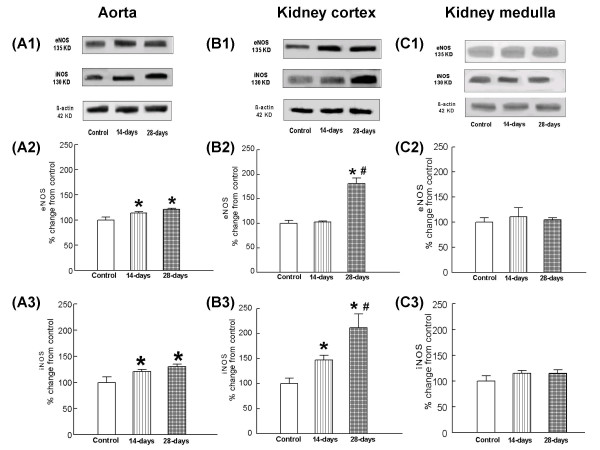
Effect of STZ-induced diabetes on the protein expression of iNOS and eNOS in thoracic aorta obtained from control, 14-days and 28-days diabetic rats. Representative protein expression of inducible- and endothelial-NOS (A1), % change in densitometric units of eNOS (A2) and iNOS (A3) in aorta, eNOS (B2) and iNOS (B3) in kidney cortex and eNOS (C2) and iNOS (C3) in kidney medulla. (N = 3 in each group); * p ≤ 0.05 Vs control group, # p ≤ 0.05 Vs 14-days diab group.

A significant up-regulation of eNOS and iNOS in thoracic aorta in 14-days and 28-days diabetes groups was obtained as compared to control group (Fig 5A1–A3). Also in kidney cortex in 28-days diabetes, eNOS and iNOS were significantly increased as compared to 14-days diabetes and control groups. Protein expression of iNOS was also significantly increased in kidney cortex in 14-days diabetes group as compared to control group (Fig 5B1–B3). In kidney medulla we did not find any significant change in eNOS and iNOS in 14-days and 28-days diabetes groups as compared to control group (Fig 5C1–C3).

### Effect of STZ-induced diabetes on the expression of total and phosphorylated p38-MAPK and ERK1/2 in thoracic aorta, kidney cortex and medulla

**Figure 6:**
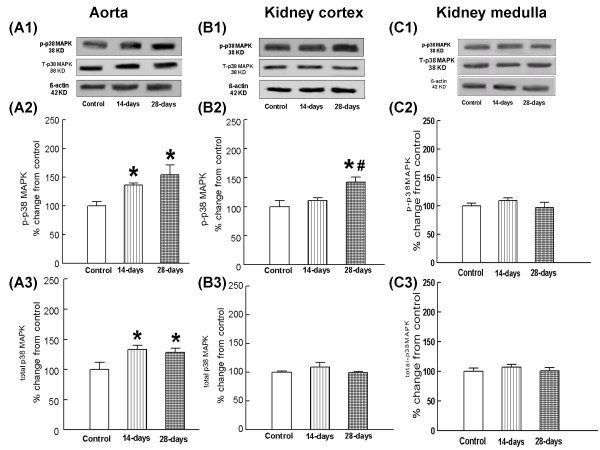
Effect of STZ-induced diabetes on the protein expression of total and phosphorylated p38-MAPK in thoracic aorta obtained from control, 14-days and 28-days diabetic rats. Representative protein expression of phospho- and total- p38-MAPK (A1, B1 and C1), % changes in densitometric units of phospho-p38-MAPK (A2, B2 and C2) and total-p38-MAPK (A3, B3 and C3) in aorta, kidney cortex and kidney medulla respectively. (N = 3 in each group); * p ≤ 0.05 Vs control group, # p ≤ 0.05 Vs 14-days diab group.

Total p38-MAPK and phosphorylated p38-MAPK protein expressions in thoracic aorta were significantly elevated in 14-days and 28-days diabetes groups as compared to control group (Fig 6A1–A3). Expression of phosphorylated p38-MAPK was significantly increased in kidney cortex obtained from 28-days diabetes group as compared to 14-days diabetes and control groups. Total p38-MAPK was not altered in kidney cortex in 14-days and 28-days diabetes groups as compared to control group (Fig 6B1–B3). In kidney medulla of 14-days and 28-days diabetes groups we did not observe any significant change in total and phosphorylated p38-MAPK (Fig 6C1–C3).

**Figure 7:**
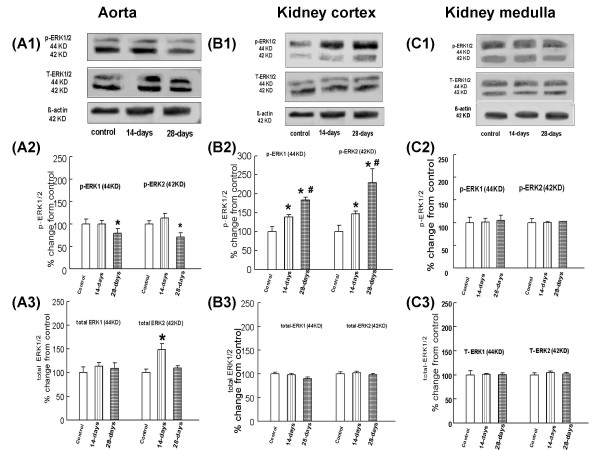
Effect of STZ-induced diabetes on the protein expression of total and phosphorylated ERK 1/2 in thoracic aorta obtained from control, 14-days and 28-days diabetic rats. Representative protein expression of phospho- and total-ERK (A1, B1 and C1), % changes in densitometric units of p-ERK (A2, B2 and C2) and Total-ERK (A3, B3 and C3) in aorta, kidney cortex and kidney medulla respectively. (N = 3 in each group); * p ≤ 0.05 Vs control group, # p ≤ 0.05 Vs 14-days diab group.

A significant decrease in the expression of phosphorylated ERK 1/2 of thoracic aorta in 28-days diabetes group but not in 14-days diabetes group was observed as compared to control group. We found that total ERK 2 but not total ERK 1 was significantly increased in thoracic aorta in 14-days diabetes groups as compared to control group. There was no change in the expression of total ERK 1 in 14-days and 28-days diabetes groups and ERK 2 in 28-days diabetes group as compared to control group (Fig 7A1–A3). In kidney cortex phosphorylated ERK 1/2 protein expression was significantly elevated in 28-days diabetes group as compared to 14-days diabetes and control groups. In kidney cortex, phosphorylated ERK 1/2 was significantly elevated in 14-days diabetes group as compared to control group. Total ERK 1/2 was not altered in kidney cortex in 14-days and 28-days diabetes groups (Fig 7B1–B3). In addition, in kidney medulla we did not observe any significant change in total and phosphorylated ERK 1/2 in 14-days and 28-days diabetes group as compared to control group (Fig 7C1–C3).

## Discussion

STZ has long been used as a drug of choice to induce *type 2 *diabetes in various animal models. This well-established model is characterized by insulin deficiency associated with insulin resistance [18]. It was reported that a single intravenous injection of STZ (55 mg/kg) could cause increased plasma glucose levels, decrease in body weight and 17% mortality in rats [18]. In the present study, too, we have observed a mortality of 20% in 14-days diabetes group and 26% in 28-days diabetes group. STZ-treated rats, post 48-h, were confirmed to be hyperglycemic, lost body weight 10% and 13% in 14-days and 28-days diabetes groups respectively. Kavalali *et al*. [19] found that food and water intake amount was higher in diabetic groups than the control group. In our study too, we observed that diabetic rats had an increase in the food intake and water intake following 14-days and 28-days of STZ-administration. These observations suggested that single intravenous injections of STZ (60 mg/kg) produced a reproducible and consistent model of diabetes in our laboratory conditions.

Renal hypertrophy can be detected as early as one day after the onset of diabetes and seen regularly post 60-hr of single STZ injection [20]. It has been reported that diabetes-induced renal hypertrophy produces increased dimensions of renal cells along with increased kidney weight [21]. In the present study, we observed an elevated kidney weight corrected by body weight in diabetic (14-days and 28-days) rats suggesting that STZ-induced diabetes produced renal hypertrophy. We also observed that both 14- and 28-days diabetic rats exhibited reduced renal blood flow along with 3-2 fold increase in plasma creatinine concentration. Umerani and Goyal [22] demonstrated an increase in serum creatinine as an indicator of deteriorated renal function in diabetic rats. Similar to our results, Itoh and coworkers [23] also demonstrated that serum creatinine levels in control group and diabetes group varied from 0.8 to 1.4 mg/dl respectively. Thus the results obtained in our study provide evidence for diabetes-induced renal dysfunction in the rat.

ET-1, a potent vasoconstrictor peptide, has been implicated in diabetes and cardiovascular disorders. Elevated, unchanged and attenuated plasma ET-1 levels have been reported during diabetes [24-27]. Although the reason for such a variation in findings appears difficult to fathom, the discrepancies in data may be attributed to differences in species of animals, duration of hyperglycemia, dose of STZ administered etc. In the present study, we observed decreased aortic and renal blood flow in both 14-days and 28-days diabetics rats. However elevation of ET-1 and NOx in plasma was seen only in 28-days diabetic rats group but not in 14 days-diabetes group. Taken together, these observations suggest that STZ-induced hyperglycemia produced alterations in the systemic and regional blood flow, which could be due to altered systemic levels of ET-1 and NO. Makino *et al*. [27] demonstrated upregulated preproET-1 mRNA in the aorta from STZ-induced diabetic rats. They suggested that increased release of ET-1 from the aorta contributes to enhanced plasma level of ET-1 seen in diabetic rats. Also ET-1 concentration has been shown to increase in kidneys [28] following diabetes. Since ET-1 exists not only in the vascular endothelial cells but also in the mesangial cells or tubular cells, this increase in ET-1 could be attributed to increased biosynthesis of ET-1 in the renal cells [28-30]. In the present study, we also observed an elevated expression of preproET-1 proteins in aorta and kidney cortex but not in kidney medulla at 14-days and 28-days following diabetes induction. These findings suggest that during early diabetes an upregulation of ET-1 biosynthesis in vascular (thoracic aorta) and locally at the organ level (kidney cortex) could be responsible for vascular and renal dysfunction seen during diabetes. Although the mechanisms of ET-1 elevation during diabetes are relatively unknown, several research groups speculated that this increase could be due to an abnormal production by the affected endothelium [31] or lack of suppression of ET-1 release secondary to attenuated endothelium-derived relaxing factor production [32].

Hirata *et al*. demonstrated that ET-1 via binding to ET_B _receptors produces activation of NOS [33] and generates NO. In the present study, elevated concentration of NOx in plasma, thoracic aorta and kidney cortex but not in medulla was observed in 28-days diabetes group. Stockklauser-Farber *et al*. [7] demonstrated an increased myocardial NOS (iNOS and eNOS) activity that reached maximal values after 4 wk and 6 wk diabetes. We speculated that along with elevated NO production, activation of NOS isoforms may play a prominent role in the pathophysiology of nephropathy at different phases of STZ-induced diabetes [34,35]. Increased expression of eNOS in afferent arterioles and glomeruli was found by Sugimoto *et al. *[36]. In a previous study, these authors demonstrated an enhanced renal expression of iNOS 5 days post diabetes that was sustained for 20 days, while eNOS and nNOS were not altered [37]. We also observed that the progression of diabetes from 14-days to 28-days upregulated iNOS and eNOS in thoracic aorta and kidney cortex while decreasing aortic and renal blood flow that correlated well with systemic and local increase in NOx levels in thoracic aorta. This suggests that NO stimulation in thoracic aorta and kidney cortex occurs with increased duration of hyperglycemia in STZ-induced diabetic rats. Since in the present study both ET-1 and NOS activation exhibit a similar course in 28-days diabetic rats, we speculate that NOS stimulation could be due to activation of ET_B _receptors via elevated ET-1 mechanisms. However, further studies will be required to strengthen this speculation.

### Signaling mechanisms in diabetes

Hyperglycemia has been shown to phosphorylate ERK1/2 in rat glomerular and mesangial cells [38] and p38-MAPK in vascular smooth muscle cells and aorta in derived from diabetic rats [39]. Pearson *et al*. [40] and Tian *et al*. [41] demonstrated that ET-1 stimulation of mesangial cell (MC) proliferation involves several pathways, among which MAPK figures prominently. In the present study, we observed that 14-days diabetes up-regulated phosphorylated p38-MAPK but not ERK1/2 in thoracic aorta. We also observed down-regulation of phosphorylated ERK 1/2 in thoracic aorta post 28-days diabetes. In kidney cortex although p38-MAPK was not altered post 14-days diabetic group, ERK1/2 is elevated. This suggests that ERK1/2 phosphorylation predominate during 14-days diabetes in kidney cortex but not in thoracic aorta, while both p38-MAPK and ERK1/2 remain unaffected in kidney medulla.

Both *in vivo *and *in vitro *results suggest that ERK and p38MAPK may be involved in high-glucose-induced cellular hypertrophy [42]. In the present study, we observed that ERK1/2 precedes p38-MAPK phosphorylation depending upon the progression of diabetes from 14-days to 28-days. We observed a profound increase in plasma NOx levels and iNOS expression with a corresponding increase in p38-MAPK activation in kidney cortex and thoracic aorta during 28-days diabetes. We propose that p38-MAPK activation could be an important signaling mechanism that causes iNOS activation as was reported by Liu *et al*. [14]. We speculate that activated p38-MAPK and iNOS mechanisms outweigh ERK1/2 mechanisms in kidney cortex and thoracic aorta during moderate diabetes. A similar finding, though not directly related to the present study, was shown by Purves *et al*. [43] where they demonstrated using cultured sensory neurons that co-treatment with high glucose and oxidative stress results in an additive effect on p38-MAPK phosphorylation without affecting ERK1/2 activation. The findings of the present study suggested that phosphorylation of p38-MAPK and not ERK1/2 was associated with iNOS activation and renal and vascular dysfunction following 28-days of STZ-induced hyperglycemia.

The results obtained in the present study characterize the STZ (60 mg/kg, I.V.)-induced diabetic rat model in our laboratory. The marked characteristics of diabetic rat model include weight loss, increase food and water intake, and bradycardia. STZ-induced diabetes, both at 14-days and 28-days, produced renal dysfunction and vascular dysfunction that correlated well with levels of ET-1 and NOx and expression of preproET-1 and NOS proteins in kidney cortex and thoracic aorta. The data obtained in the present study demonstrate that progression of diabetes from 14-days to 28-days caused a factorial increase in p38-MAPK phosphorylation along with NOS upregulation in kidney cortex and thoracic aorta. The study provides evidence that diabetes produces vascular and renal dysfunction with a profound effect on signaling mechanisms at later stage of diabetes. However, more studies will be required to further delineate the inherent link or interaction between p38-MAPK upregulation, ET-1 and NO mechanisms and development of renal and vascular dysfunction during diabetes.

## Figures and Tables

**Table 1:** Effect of STZ-induced diabetes on percent body weight (BW) change, blood glucose levels (mg.dL^-1^), mean arterial pressure (mmHg), heart rate (beats per min), kidney weight/100 g BW, and plasma creatinine concentration (g.dL^-1^.kg^-1 ^BW) at 14-days (Diab, 14-days) and 28-days (Diab, 28-days). (N = 6 in each group) * p ≤ 0.05 Vs control group.

**Parameter**	**Control**	**Diab (14-days)**	**Diab (28-days)**
BW change (%)	10 ± 3	-10 ± 3*	-13 ± 2*
Blood glucose (mg.dL^-1^)	84 ± 2	480 ± 3*	486 ± 11*
Mean arterial pressure (mmHg)	130 ± 3	120 ± 6	117 ± 2
HR (Beats per min)	419 ± 5	326 ± 7*	339 ± 10*
Kidney weight/100 g BW	0.72 ± 0.03	1.1 ± 0.05*	1.0 ± 0.03*
Plasma creatinine (mg.dL^-1^.kg^-1^)	2.4 ± 0.5	6.4 ± 3.5	4.5 ± 3.5
